# Targeted exome sequencing identified a novel *USH2A* mutation in a Chinese usher syndrome family: a case report

**DOI:** 10.1186/s12886-020-01711-7

**Published:** 2020-12-10

**Authors:** Dongjun Xing, Huaiyu Zhou, Rongguo Yu, Linni Wang, Liying Hu, Zhiqing Li, Xiaorong Li

**Affiliations:** Tianjin Key Laboratory of Retinal Functions and Diseases, Tianjin International Joint Research and Development Centre of Ophthalmology and Vision Science, Eye Institute and School of Optometry, Tianjin Medical University Eye Hospital, 251 Fukang Road, Tianjin, 300384 China

**Keywords:** Usher syndrome, Targeted exome sequencing, Mutation, Case report

## Abstract

**Background:**

Usher syndrome is a disease with a heterogeneous phenotype and genotype. Our purpose was to identify the gene mutation in a Chinese family with Usher syndrome type 2 and describe the clinical features.

**Case presentation:**

A 23-year-old man complained of a 10-year duration of nyctalopia and a 3-year decline in visual acuity of both eyes accompanied by congenital dysaudia. To clarify the diagnosis, the clinical symptoms were observed and analysed in combination with comprehensive ophthalmologic examinations as well as genetic analysis (targeted exome sequencing, TES). A typical clinical presentation of Usher syndrome of the fundus was found, including a waxy yellow-like disc, bone-spicule formations and retinal vessel stenosis. Optical coherence tomography (OCT) and optical coherence tomography angiography (OCTA) showed loss of the ellipsoid zone and a reduction in paracaval vessel density in both eyes. Genetic analysis identified a novel homozygous c.8483_8486del (p.Ser2828*) mutation in *USH2A*. The mutation resulted in premature termination of translation and caused the deletion of 19 fibronectin type 3 domains (FN3), transmembrane (TM) region and PDZ-binding motif domain, which play an important role in protein binding. After combining the clinical manifestations and genetic results, the patient was diagnosed with Usher syndrome type 2.

**Conclusion:**

We found a novel c.8483_8486del mutation in the *USH2A* gene through TES techniques. The results broaden the spectrum of mutations in Usher syndrome type 2 and suggest that a combination of clinical information and molecular diagnosis via TES could help Usher syndrome patients obtain a better diagnosis.

**Supplementary Information:**

The online version contains supplementary material available at 10.1186/s12886-020-01711-7.

## Background

Usher syndrome (USH) is an autosomal recessive disease that is characterized by retinitis pigmentosa (RP), sensorineural hearing impairment and vestibule dysfunction. The prevalence of USH is approximately 3.2 to 6.2 per 100,000 individuals [1–4], and it is clinically and genetically heterogeneous. To date, 18 genes and loci have been associated with USH (RetNet [https://sph.uth.edu/retnet]; August 2020). Among them, 15 genes were identified as causative genes. USH can be divided into three types according to the age of onset, the severity of visual and hearing impairment and vestibule dysfunction. However, because the genetic manifestations are currently not well understood, the rate of missed diagnosis (4%) is high in Asia, especially in China [5].

Deafness occurs early in patients with USH1, and their abnormal visual function is easily ignored. In patients with USH2 and USH3, visual function and hearing abnormalities are gradually progressive. Accurate clinical and molecular diagnoses are the basis of prognosis prediction, treatment selection and genetic counselling. Targeted exome sequencing (TES) provides a new opportunity to reveal the genetic defects in USH patients [6]. Here, we screened 381 inherited retinal disease (IRD)-related genes in an USH2 family and identified a novel c.8483_8486del (p.Ser2828*) mutation in the *USH2A* gene.

## Case presentation

A 23-year-old man visited our clinic and had suffered from deafness from childhood with occasional dizziness, nyctalopia for 10 years and visual acuity decline of both eyes for nearly 3 years. Previously, he was diagnosed with “sensorineural deafness” by an otorhinolaryngologist. The patient and his family members gave informed consent for the study, which was approved by the Ethics Committee of Tianjin Medical University Eye Hospital (Tianjin, China). Then, peripheral venous blood samples were collected for TES and Sanger sequencing. For the clinical diagnosis, we performed a comprehensive ocular examination that included determination of best-corrected visual acuity (BCVA), slit-lamp examination, visual field tests, optical coherence tomography (OCT), optical coherence tomography angiography (OCTA), ultra-wide field fundus photography, and fundus autofluorescence (FAF).

## Methods

### DNA library preparation

Genomic DNA was extracted from peripheral blood leukocytes of the patient and his family members using a DNA Extraction Kit (TIANGEN, Beijing, China) following the manufacturer’s instructions. The DNA was quantified with a Nanodrop 2000 (Thermal Fisher Scientific, DE). A minimum of 3 μg of DNA was used for the indexed Illumina libraries according to the manufacturer’s protocol (My Genostics, Inc., Beijing, China). DNA fragments with sizes ranging from 350 bp to 450 bp and those including the adaptor sequences were selected for the DNA libraries.

### Targeted gene capture and sequencing

Next, 381 known genes associated with IRDs, including USH (Additional file 2: Table S2), were selected by a gene capture strategy using the GenCapCustom Enrichment Kit (My Genostics Inc., Beijing, China) following the manufacturer’s protocol. The biotinylated capture probes were designed to tile all of the exons with non-repeated regions. Sequencing was performed on an Illumina HiSeq 2000 sequencer (Illumina, San Diego, CA, USA) for paired-end reads of 150 bp.

### Bioinformatics analysis

Following sequencing, raw image files were processed using Bcl2Fastq software (Bcl2Fastq, Illumina, Inc.) for base calling and raw data generation. Low-quality variations (score ≥ 20) were filtered out. The clean reads were then aligned to the reference human genome using Short Oligonucleotide Analysis Package (SOAP) aligner software (SOAP2.21; soap.genomics.org.cn/soapsnp.html) (hg19). After removing polymerase chain reaction (PCR) duplicates using the Picard program, single nucleotide polymorphisms (SNPs) were determined using the SOAP SNP program, and the deletions and insertions (InDels) were detected using Genome Analysis Toolkit software 3.7. Subsequently, we annotated the identified SNPs and InDels with the Exome-assistant program (http://122.228.158.106/exomeassistant) and viewed the short read alignment using MagicViewer to confirm the candidate SNPs and InDels. Non-synonymous variants were evaluated for pathogenicity using the Sorting Intolerant From Tolerant [SIFT; (http://sift.jcvi.org/)] and PolyPhen (http://genetics.bwh.harvard.edu/pph2/) tools as well as Protein Analysis Through Evolutionary Relationships (PANTHER; www.pantherdb.org) and Pathogenic Mutation Prediction (Pmut; http://mmb.pcb.ub.es/PMut/).

### Expanded validation and protein function prediction

Genomic DNA of the proband was subjected to TES. Filtered candidate variants identified by an Illumina HiSeq 2000 sequencer were confirmed by Sanger sequencing. The coding exons containing the detected mutations were amplified using Ex Tag DNA polymerase (Takara, Dalian). The purified PCR samples were sequenced using an ABI PRISM 3730 genetic analyser (Applied Biosystems; Thermo Fisher Scientific, Inc.), and then sequence traces were analysed with Mutation Surveyor (Softgenetics, PA). The mutation was confirmed in the family members by the same procedure. Multiple sequence alignments were performed using ClustalW2 with the default setting (http://www.ebi.ac.uk/Tools/clustalw2/). Protein structures were determined by SMART (http://smart.emblheidelberg.de). The variation in the 3D structure of the protein caused by gene mutation was analysed using Protein Data Bank (PDB) and the homology modelling software Swiss-Model. All genomic DNA samples were collected after obtaining informed consent.

## Results

### Clinical findings

**Fig. 1:**
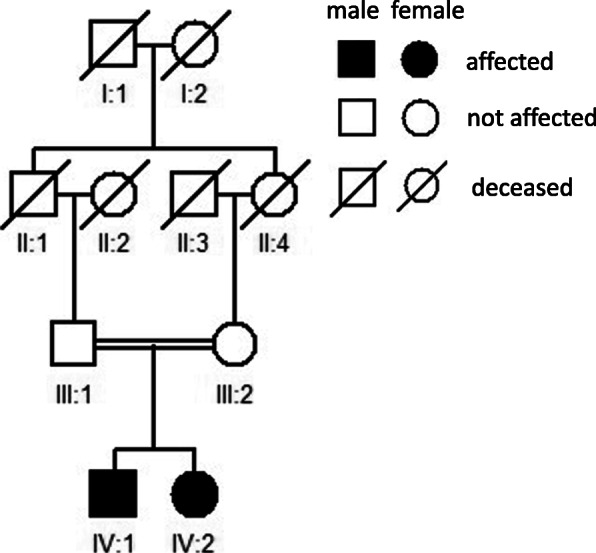
Pedigree of the family

**Fig. 2:**
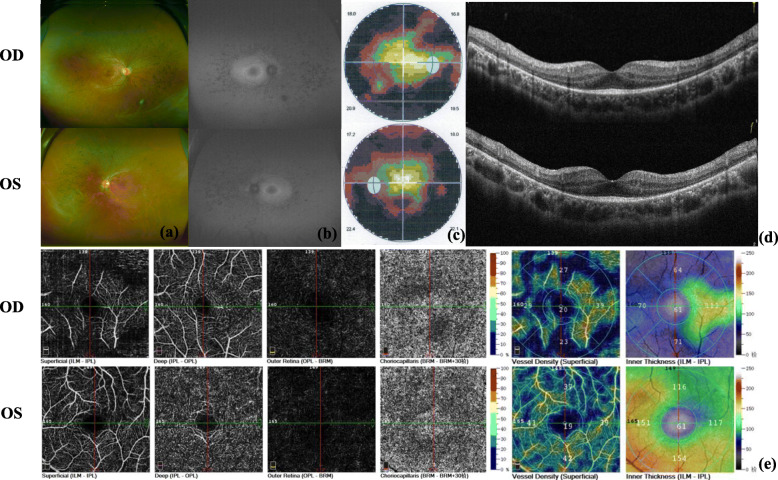
Ultra-wide-angle images of the patient demonstrated midperipheral retinal atrophy, pigment migration and attenuated retinal vessels (**a**). FAF demonstrated midperipheral patchy hypoautofluorescence with a parafoveal autofluorescent ring (**b**). The visual field of the patient detected by the Octopus perimeter device demonstrated constriction in each eye (**c**). OCT demonstrated loss of the ellipsoid zone consistent with atrophy of the outer nuclear layer outside the hyperautofluorescent ring on FAF (red line) (**d**). Macular OCTA images demonstrated an enlarging FAZ in both the superficial capillary plexus and deep capillary plexus. Macular vascular flow density (superficial) was decreased in both eyes (**e**)

A 23-year-old man presented a 10-year history of deafness and poor night vision. His BCVA was 0.6/1.0 (R/L). His parents had a consanguineous marriage, and his grandparents were deceased, but they were healthy according to their past medical history and eye conditions. In addition, his parents and his sister were unaffected (Fig. 1). Ophthalmologic investigations were performed to better clarify the proband’s condition. Slit-lamp examination showed that the anterior segment of the eyes was normal. The fundus had typical RP characteristics, including the appearance of a waxy yellow-like disc, a large amount of osteoblast-like pigmentation, and tapering of the retinal vessels, which was obvious in the arteries (Fig. 2a). An abnormal parafoveal ring of increased autofluorescence was observed in ultra-wide-angle images, and a ring-like hypoautofluorescence region was observed around the macula and optic disc on FAF imaging (Fig. 2b). Examination with an Octopus perimeter device showed a tubular visual field in both eyes (Fig. 2c). We also found decreased retinal thickness and absence of the ellipsoid zone in the macula (Fig. 2d). Macular OCTA revealed an enlarging foveal avascular zone (FAZ) in the superficial capillary plexus and deep capillary plexus, while macular vascular flow density was also decreased (Fig. 2e).

### Genetic and molecular analysis

**Fig. 3:**
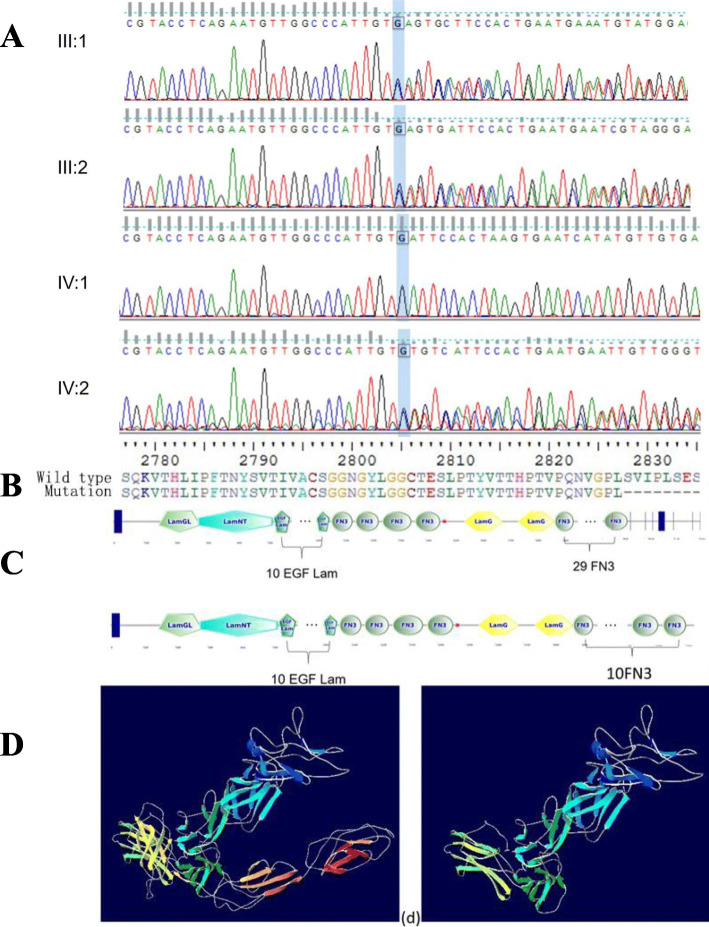
Identified mutation confirmed by Sanger sequencing. IV:1 (the patient) harboured a homozygous mutation (c.8483_8486del/c.8483_8486del), III1, III2 and IV:1 were identified with a heterozygous mutation (c.8483_8486del/−) (**a**). The amino acid sequence of the mutant usherin (**b**). The tertiary structure of usherin; 19 FN3, TM and PDZ-binding motif domains were lost in the truncated protein (**c**). The change in the folded state of the protein (**d**)

DNA extracted from the peripheral blood was subjected to TES (Additional file 1: Table S1). Genetic tests showed that the patient had a novel mutation (c.8483_8486del) in the USH2A gene. Moreover, DNA samples extracted from the proband’s sister and parents were used for Sanger sequencing. Genetic co-segregation analysis was confirmed in this family (Fig. 3a). A model structure for USH2A was generated via homology modelling (Fig. 3b). The mutation resulted in premature termination of translation, and the stop-gain variant was predicted to remove 2375 amino acids from the encoded protein, which would result in truncation of the α/β-hydrolase domain (Fig. 3c) and may change the overall function of the folded state of the protein (Fig. 3d).

## Discussion and conclusions

We reported the case of a 23-year-old patient who presented a series of typical clinical features with a novel homozygous mutation, p.Ser2828* (rs1199684717), in USH2A, a gene responsible for USH2 (OMIM:276901). The frequency of the mutation is 0.000004 in the Genome Aggregation Database, and it was found in a heterozygous state in one European non-Finnish individual. Mutations in *USH2A* are associated with USH2, which is responsible for almost 50% of USH cases [7].

*USH2A* codes two alternatively spliced isoforms of usherin. The short ~ 170 kDa isoform a, consisting of 21 exons, is regarded as an extracellular protein. The full-length ~ 580 kDa isoform b is a complex transmembrane protein composed of three regions: a large extracellular region consisting of an N-terminal signal peptide, laminin G-like domain (LamGL), laminin domain N-terminal (LamNT), laminin-type EGF-like modules (EGF-Lam), fibronectin type III (FN3) repeats, laminin G domains (LamG); a transmembrane region (TM); and a cytoplasmic C-terminal domain containing a PDZ-binding motif [8, 9]. Usherin is distributed in the periciliary membrane complex and synapse in photoreceptors. All USH1 and USH2 proteins are organized as protein networks by the scaffold proteins harmonin (USH1C), whirlin (USH2D) and SANS (USH1G). Usherin (*USH2A*) and VLGR1b (*USH2C*) are part of the links that are intracellularly attached to the scaffold proteins. However, during the differentiation of the hair bundle, both USH1 and USH2 proteins contribute to the formation of side links located at the tip and the base of the stereocilia, respectively. They exist in multiprotein complexes that work together as molecular networks to anchor them to the stereocilia actin filaments [10–14].

The homozygous mutation (p.Ser2828*) in *USH2A* caused premature termination of translation, and as a result, 19 FN3, TM and PDZ-binding motif domains were deleted. FN3 plays a key role in cell adhesion, cell morphology, thrombosis, cell migration, and embryonic differentiation as well as pathophysiologic processes such as angiogenesis and vascular remodelling [15]. A TM domain is present at the base of differentiating stereocilia and causes the mechanosensitive hair bundles to be receptive to sound. PDZ-binding motif domains provide the anchoring of interstereocilia lateral links to the F-actin core of stereocilia [16]. In this regard, we suppose that the absence of these domains corresponding to the incompleteness of usherin might have affected the process of differentiation and maturation of the stereocilia, resulting in a milder dysmorphic phenotype of the stereocilia. Several missense hotspots have been associated with the pathogenesis of FN3 in usherin [17], which supports our hypothesis. However, this pathway needs to be confirmed by molecular experiments in the future.

Whole-genome sequencing (WGS), whole-exome sequencing (WES) and TES are three major methodologies for molecular diagnosis of IRDs. WGS is useful for detecting copy number and structural variations [18]. WES is especially useful for identifying novel IRD-related genes. TES is an accurate, rapid and cost-effective approach for screening of multiple genes [19], but it still has some major limitations, such as detecting variants in low-depth regions and copy number variations [18, 20]. Because of their high cost, both of WGS and WES are less widely used than TES. TES is suitable for molecular diagnosis of USH. Because of the great diversity of various types of pathogenic genes and the frequent occurrence of new mutations, array-based diagnosis often can not accurately reflect pathogenicity. Pathogenic USH genes have many subtypes and numerous exons. At present, more than 400 coding exons have been identified [21]. Therefore, a higher diagnosis rate can be obtained using a sequence-based diagnosis method.

Here, we report a novel homozygous mutation, c.8483_8486del, in the *USH2A* gene identified through TES techniques. The mutation truncated *USH2A* gene translation, and 19 FN3, TM and PDZ-binding motif domains were lost, which influenced the function of stereocilia. We broadened the spectrum of mutations of this disease and provided a new locus for gene therapy of USH. The combination of molecular diagnosis by TES and clinical information can help USH patients obtain more accurate diagnoses.

## Supplementary Information

**Additional file 1: Table S1.** 381 known genes associated with IRD.

**Additional file 2: Table S2.** The TES data of the proband.

**Additional file 3.**

**Additional file 4.**

**Additional file 5.**

**Additional file 6.**

## Data Availability

All data are fully available without restriction. The TES data of the proband in Additional file 1: Table S1; The data of Sanger sequencing: III1, III2, IV1, IV2.

## Abbreviations

USH: Usher syndrome
OCT: Optical coherence tomography
OCTA: Optical coherence tomography angiography
IRD: Inherited retinal disease
FN3: Fibronectin type 3
TES: Targeted exome sequencing
RP: Retinitis pigmentosa
BCVA: Best-corrected visual acuity
FAF: Fundus autofluorescence
SOAP: Short Oligonucleotide Analysis Package
SNPs: Single nucleotide polymorphisms
PCR: Polymerase chain reaction
PDB: Protein Data Bank
FAZ: Foveal avascular zone
LamGL: Laminin G-like domain
LamNT: Laminin domain N-terminal
EGF-Lam: Laminin-type EGF-like modules
LamG: Laminin G domains
